# AI deepfake: GPT-4o can produce near-authentic fundus images

**DOI:** 10.1038/s41433-025-03937-5

**Published:** 2025-07-19

**Authors:** Andrea Taloni, Maurizio Taloni, Giulia Coco, Danson V. Muttuvelu, Massimo Busin, Giuseppe Giannaccare

**Affiliations:** 1 Department of Translational Medicine, University of Ferrara, Ferrara, Italy; 2 Department of Ophthalmology, Ospedali Privati Forlì “Villa Igea”, Forlì, Italy; 3 Istituto Internazionale per la Ricerca e Formazione in Oftalmologia, Forlì, Italy; 4 Osteo-odonto-keratoprosthesis Foundation, Rome, Italy; 5 Department of Clinical Sciences and Translational Medicine, University of Rome Tor Vergata, Rome, Italy; 6 Faculty of Health and Medical Sciences, University of Copenhagen, Copenhagen, Denmark; 7 MitØje v/ Danske Speciallæger, Aarhus, Denmark; 8 Eye Clinic, Department of Surgical Sciences, University of Cagliari, Cagliari, Italy

**Keywords:** Technology, Medical research

On March 25th, 2025, OpenAI announced “ChatGPT-4o Image Generation”, a new text-to-image generator integrated into the large language model (LLM) GPT-4o. The model introduced groundbreaking innovations, offering enhanced prompt adherence and photorealism [1]. LLMs traditionally struggled to interpret and produce ophthalmological images [2]. As soon as ChatGPT-4o Image Generation became available on our ChatGPT Plus subscription account (March 26th), we investigated whether the new model could allow the generation of realistic ophthalmological images.

Before opening a new GPT-4o chat session, the ChatGPT Memory feature was disabled to avoid potential influence from previous conversations. We prompted the model to “generate a realistic image of a healthy retinal fundus photograph of the posterior pole” (Fig. 1). Although the output image appeared authentic at first glance, a deeper examination revealed hints of fabrication. Most notably, the retinal background was excessively homogeneous, lacking any sign of choroidal vascular patterns. In addition, the course of blood vessels was atypical, displaying unnatural crossings (*), marked axial light reflex (†), and sudden changes in caliber (‡).

**Fig. 1:**
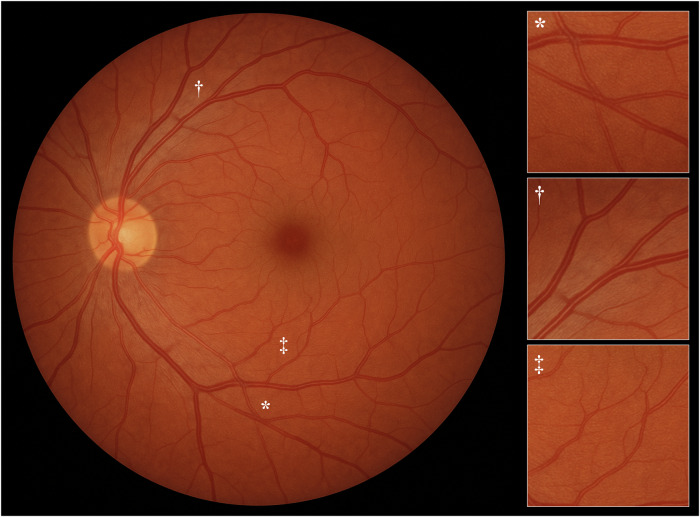
Retinal fundus photograph generated by ChatGPT-4o image generation with the prompt “generate a realistic image of a healthy retinal fundus photograph of the posterior pole”. The panels on the right provide magnified views of areas featuring hints of fabrication: unnatural crossings (*), marked axial light reflex (†), and sudden changes in caliber (‡).

To attempt to enhance realism, we uploaded a real fundus image to GPT-4o, along with a prompt to “generate a fundus photograph as similar as possible to this one”. The authentic fundus shot was captured from a healthy 49-year-old woman using the Digital Fundus Camera Canon CR-2 (Canon Medical Systems Corp., Otawara, Tochigi, Japan) (Fig. 2A). Generative AI cannot provide exact replicas of uploaded images; however, they can be used as reference to produce similar outputs. The new fundus photograph generated by GPT-4o was more realistic than the previous one (Fig. 2B). Choroidal vasculature was present, and retinal vessels, although still exhibiting a pronounced axial light reflex, appeared compatible with normal retinal anatomy. The optic disc cup was smaller compared to the authentic fundus image.

**Fig. 2:**
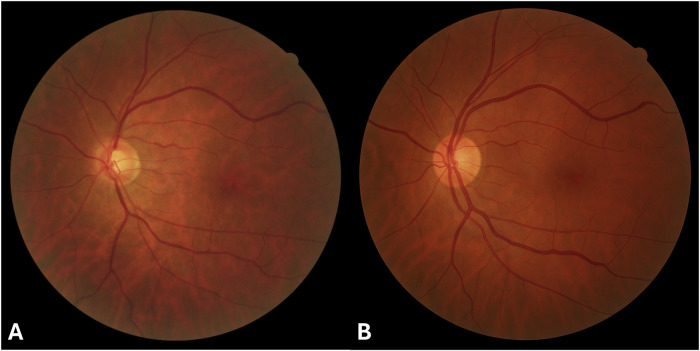
Creating AI-generated fundus photographs using real fundus images. On the left, authentic fundus photograph uploaded to ChatGPT-4o Image Generation along with the prompt “generate a fundus photograph as similar as possible to this one” (**A**). On the right, synthetic fundus photograph generated by ChatGPT-4o using the authentic photograph as reference (**B**).

Our prompts did not include specific instructions regarding patient’s age, the appearance of the macula, optic disc, or retinal vasculature. Alternative prompting strategies may be explored to further improve realism.

Deep learning models have been used in ophthalmology for the detection, classification, and grading of retinal diseases. However, training AI models requires extensive datasets of images. To overcome this limitation, researchers developed generative adversarial networks (GANs) that can synthesize high-resolution images aimed at augmenting real image datasets [3–5]. Burlina et al. proposed several criteria for synthetic fundus images to be suitable for inclusion in training datasets. First, realism should be sufficient to enable retinal specialists to reliably diagnose and grade diseases. Specialists should be unable to distinguish synthetic images from real ones. Additionally, deep learning algorithms trained on synthetic images should achieve performance comparable to those trained on real datasets. Finally, images should exhibit sufficient variability and be distinguishable from one another [3].

Developing GANs requires technical expertise and substantial computational resources, while LLM-based image generation may offer a faster, cheaper alternative. This is the first report to demonstrate that a publicly accessible LLM can generate high-resolution, authentic-looking retinal photographs. Further research is needed to determine whether such images can be used in training datasets.

## Data Availability

A.T. had full access to all the data in the study and takes responsibility for the integrity of the data and the accuracy of the data analysis.
